# Recommendations on service delivery to help reduce suffering and anxiety in patients and caregivers post-hematopoietic cell transplantation: a case report

**DOI:** 10.1186/s13256-021-03126-4

**Published:** 2021-11-05

**Authors:** Jaleel Mohammed, Hadeel R. Bakhsh, Cliodhna Craig, Shahrukh K. Hashmi

**Affiliations:** 1 Lincolnshire Community Health Services NHS Trust, Lincoln, LN5 7JH UK; 2 Rehabilitation Association for Hematopoietic Cell Transplantation, Gloucester, UK; 3 Department of Rehabilitation, College of Health and Rehabilitation Sciences, Princess Nourah Bint Abdulrahman University, Riyadh, Saudi Arabia; 4 Department of Internal Medicine, Mayo Clinic, Rochester, MN USA; 5 Sheikh Shakhbout Medical City, Abu Dhabi, UAE

**Keywords:** Occupational therapy, HSCT, Rehabilitation, Graft-versus-host disease, Case report

## Abstract

**Background:**

The aim of this study is to highlight the importance of having a central case managing team and to make some strong recommendations that can have a positive impact on the lives of hematopoietic stem cell transplantation survivors.

**Case presentation:**

A 2-year-old white child who was diagnosed with acute myeloid leukemia and underwent hematopoietic stem cell transplantation in May 2014 relapsed in March 2017, and underwent a second hematopoietic stem cell transplantation in July 2017, at which point he suffered from graft-versus-host disease. This case report presents his journey and that of his caregivers, and the challenges they faced as patient and parents in pursuit of optimal quality of life during the survivorship period. The case study emphasizes not only the challenges faced by patients but also identified gaps in post-hematopoietic cell transplantation care service delivery. Furthermore, the case study also highlights the importance of involving caregivers in post-transplant care and having a better communication process and service facilitation process throughout the journey of the patient and their carer.

**Conclusions:**

Transplant centers have a duty of care, and a proactive approach with a well-defined pathway is needed for managing post-transplant complications and reducing stress and anxiety for patients and their caregivers.

**Supplementary Information:**

The online version contains supplementary material available at https://doi.org/10.1186/s13256-021-03126-4.

## Introduction

Multiorgan involvement after hematopoietic stem cell transplantation (HSCT) in patients with graft-versus-host disease (GVHD) is not a new phenomenon, and it has been reported in literature for several decades [1]. Physical disabilities [2, 3], poor quality of life [4], psychological impact [5], and increased mortality associated with GVHD are well documented and acknowledged by transplant clinicians worldwide [6]. Due to such diverse organ involvement, several organizations, including the Haemato‐oncology subgroup of the British Committee for Standards in Haematology and the British Society for Bone Marrow Transplantation, put forward recommendations on the importance of a multidisciplinary approach and involvement of organ-specific specialists for managing post-HSCT complications [7].

Unfortunately, in reality, an increasing number of patients are missing out on many vital services, and the majority of patients undergoing HSCT globally do not have any structured multidisciplinary pathway or referral system in place. Referral to specialist services so far seems to be a reactive response to already developed symptoms, and the availability of services to this patient group differs from one National Health Service policy to another, and is dependent on the awareness and training level of the staff about the disease complexity and the availability of specialist services in the area [8]. The patient and their caregivers often must connect the dots and sometimes pass on from one service to another without any specific plan or communication between the various specialties.

Here we present a case report of a child who suffered from GVHD, his journey and that of his caregivers, and the challenges they faced as a patient and parents in pursuit of optimal quality of life during the survivorship period. Through this story, we highlight the importance of having a central case managing team and make strong recommendations that we believe can have a positive impact on the lives of thousands of HSCT survivors.

## Case presentation

A white British boy was diagnosed with acute myeloid leukemia at the age of 2 years. He underwent HSCT in May 2014, relapsed in March 2017, and underwent a second HSCT in July 2017. Aside from acute infections (Epstein–Barr virus and adenovirus), the child had no manifestations of acute or chronic GVHD following the first HSCT transplant in May 2014. However, his T cells dropped early post-transplant, requiring immediate withdrawal of ciclosporin and subsequent donor lymphocyte infusion.

### Interventions and outcomes

Following the second HSCT transplant in July 2017, the acute GVHD manifestations were so severe that he was hospitalized for almost 7 months and was under a conditioning regimen post-second transplant outlined in Table 1. Consequently, the child experienced the following outcomes: mucositis requiring total parenteral nutrition (TPN); posterior reversible encephalopathy syndrome attributed to ciclosporin, seizures D + 16 requiring brief pediatric intensive care unit (PICU) admission; bronchiolitis obliterans; hypertension; sinus tachycardia; hemorrhagic cystitis—BK virus positive; adenovirus in pharyngeal and nasal swab; blood culture—*Enterococcus faecium* septicemia; right ear discharge—*Staphylococcus aureus*; and influenza B in nasal swab (symptomatic).

**Table 1:** Conditioning regimen

Drug	Dose	Days	Cumulative dose
Fludarabine intravenous	30 mg/m^2^/day	D-6 to D-2	150 mg/m^2^
Treosulfan intravenous	14 mg/m^2^/day	D-6 to D-4	42 g/m^2^
Thiotepa intravenous	5 mg/kg BD	D-3	10 mg

mg, millgram; m^2^, square meter; kg, kilogram; BD, twice a day

In addition to the above mentioned effects, the child developed grade IV GVHD that manifested in several organs as follows:

#### Skin

Engraftment/immunological fevers at D + 11 with rashes.

#### Liver

Bilirubin rose D + 29, initially remaining under 100 mg/dL, but, from D + 40, this increased to 299–411 mg/dL along with alkaline phosphatase (ALP) (700–815 U/L) and alanine aminotransferase (ALT) (100–140 U/L). He also had raised gamma-glutamyl transferase (GGT).

#### Eyes

Ophthalmology review D + 42 confirmed corneal abrasions in bilateral eyes requiring repeated membrane debridement under general anesthesia and corneal lens insertion to aid healing in the eyes.

#### Lungs

Widespread lung crepitations and increased work of breathing unresponsive to anti-infectives attributed to bronchiolitis obliterans developing from lung GVHD. Chest computed tomography (CT) scan also showed pneumomediastinum.

#### Gut

Ileus and bile-stained aspirates D + 105 revealed GVHD exacerbated by intercurrent infections of the bowel with persistent bowel wall thickening. D + 122 showed inflammation in the duodenum and stomach, but with a fairly normal colon with occasional patches of mild inflammation.

#### Nutrition

The child was on TPN with 21% concentration of E028 for the entire time he was hospitalized. Nasogastric feed was attempted on numerous occasions, starting with the most basic, predigested form of feed at 1 mL per hour. However, this was stopped until the caregivers were able to build up nasogastric nutrition upon hospital discharge.

#### Musculoskeletal

Upon weaning of his sixth pulse of methylprednisolone and the subsequent hydrocortisone (May/June 2018), the child’s skin and soft tissue condition deteriorated to the extent that he lost all his independence and mobility (Table 2). There were signs of deterioration, that is, daily temperatures, shuffling along, stiffening, low mood, poor appetite, random edema, and platelet count of 700,000 µL (which the parents were told was a sign of well-working bone marrow and felt reassured by this). In July 2018, a magnetic resonance imaging (MRI) scan confirmed the swelling on the neck as thickening of the soft tissue, and there were blisters on his arms. A follow-up MRI scan in February 2019 reported bone marrow, muscle, subcutaneous tissue, and intermuscular/fascial edema, particularly around the shoulder girdles and pelvis with probable bursal fluid collections around the subscapularis and iliopsoas muscles.

**Table 2:** Joint range of motion scores

Joint	Left	Right
Knee extension	−60°	−40°
Shoulder flexion	50°	55°
Elbow extension	−90°	−90°
Elbow supination	1/4	1/3
Finger/thumb opposition	To index finger	To fourth finger

Moreover, another follow-up MRI scan in June 2019 served as a comparison with the prior study, and the findings demonstrated the development of pleural effusions. The pattern and extent of joint effusions and soft tissue edema remained unchanged. Overall, appearances were consistent with inflammatory changes, albeit nonspecific (Additional file 1: Appendix S1).

**Fig. 1:**
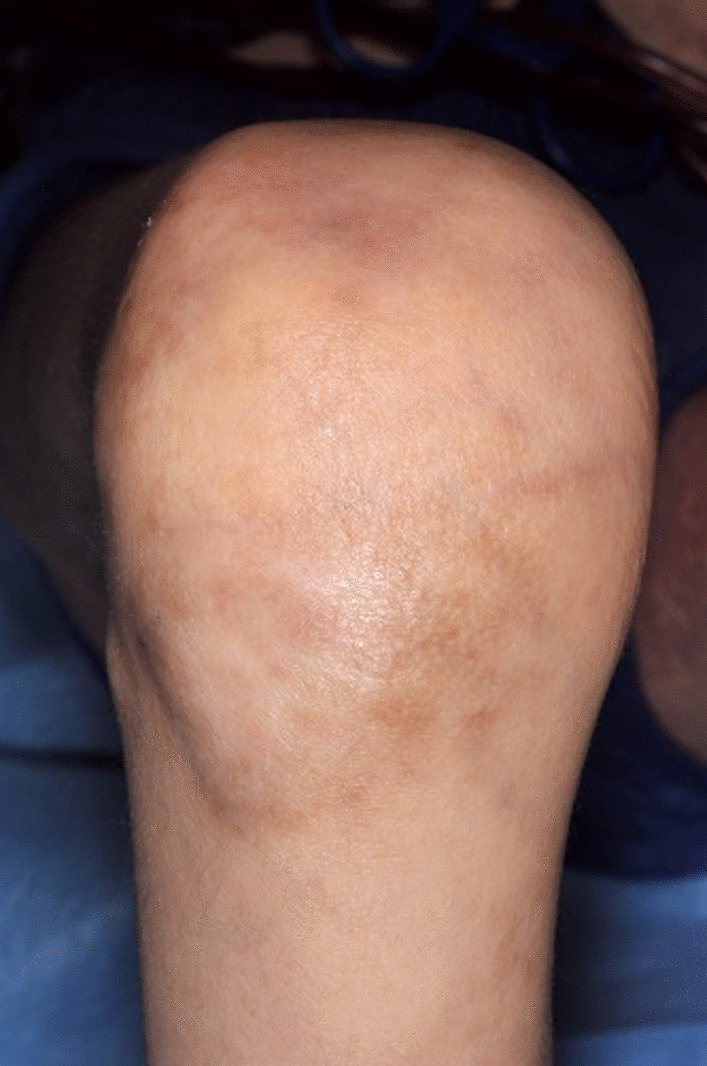
Knee contracture

**Fig. 2:**
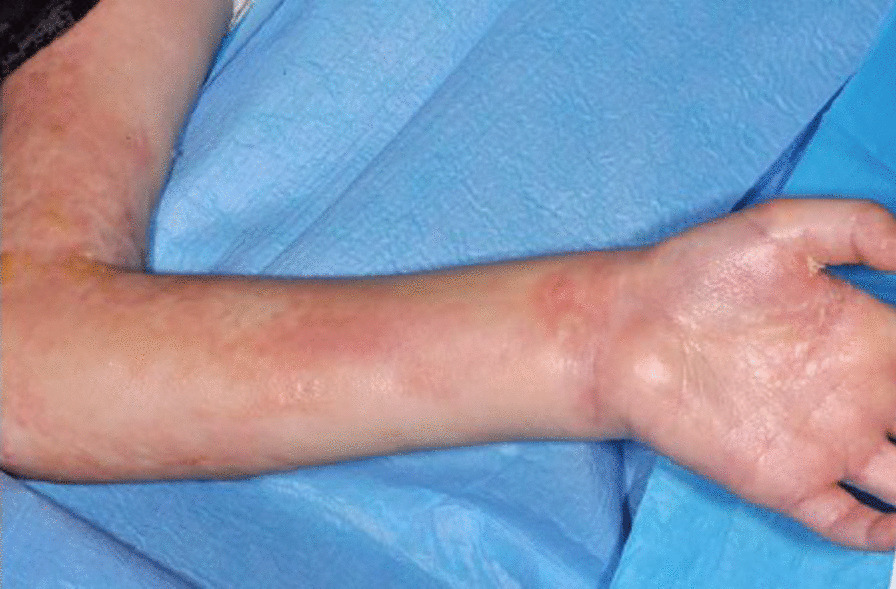
Elbow contracture

The physical assessment results from June 2019 included the modified Rodnan skin score of 36/51 and passive range of motion (P-ROM) score for shoulder of 2 (1–7), elbow 4 (1–7), wrist/fingers 2 (1–7), and ankle 2 (1–4), with an overall score of 3. This indicates contractures with a significant decrease in range of motion and significant limitation in the activities of daily life (Additional file 2: Appendix S2, Additional file 3: Appendix S3). Daily physiotherapy and splinting served only to prevent the joints from becoming more contracted as opposed to improving the range of movement (Figs. 1 and 2).

### Medication

In July 2018, almost a year post-transplant and after the development of the musculoskeletal manifestations of the illness, the parents were keen to consider new medication, given that he was clearly steroid refractory. The push for new medications took a phenomenal amount of research and worldwide conversations led by the parents. The child was on the drug imatinib, but after 1 week he was admitted to the intensive care unit (ICU) and 5 pounds of fluid was removed from his body. The drug was immediately withdrawn.

Ibrutinib initially had a strong partial response, in that his platelet count normalized from 700,000+ µL, and his liver enzymes and C-reactive protein (CRP) also normalized. There was temporary sporadic softening of the soft tissue. At the peak, 3 months into ibrutinib, he was cycling on an adapted bike and bravely undertaking 10–15 second runs, albeit with considerable effort. The downsides were further waning of appetite and sporadic cramping.

Throughout his illness, the child had eight pulses of methylprednisolone, five courses of mesenchymal stromal cells, and extracorporeal photopheresis for 2 years. He received imatinib, ibrutinib, rituximab, tacrolimus, autologous serum eye drops, and antimicrobials. He wore day and night splints, used a spirometer, and used Aerobika for pulmonary exercises. He also tried using silk, custom-made gloves for the scarring on his hands, silicone patches on the knee and elbow joints, and K-tape on his soft tissue; the latter two were abandoned due to changes in the skin. Ruxolitinib was considered as the next step, but time ran out. The parents considered using, but ruled out, cannabis oil, oxygen chambers, gene therapy, and saline tanks on the advice of medics.

### Post-hospital discharge

His forced expiratory volume in the first second (FEV1) never surpassed 28%, although his bilirubin and other liver enzyme levels normalized. He was on nighttime oxygen, 0.5 L nasal prongs, and fluticasone, azithromycin, and montelukast (FAM) therapy. His eyes, gut, and skin were all stable, and physiotherapy was irregular, focusing on conditioning after prolonged hospitalization. Moreover, he was eating normally by then but struggled to achieve a balanced diet with the calorie intake required for his age and condition, which is an average of 1000 kcal per day with significant effort. Both nasogastric and TPN were absent, and consultants gave mixed advice regarding his nutrition.

#### Psychological impact of treatment journey

The child’s psychological state was devastating for a young child, in time telling his family “he needed to die” and how “this was killing him.” Due to lack of education and guidance to the family on the changes in their child, the anxiety and stress on the entire family, who were in a 24/7 care-giving mode, were immense. The advice from various healthcare professionals was to “do yoga as he was on all the right medicines,” leaving the family to believe that they would stretch their way out of the situation. Knowledge and advice on the type of splints that could help counteract the severe contractures in all body joints, particularly the arms and legs, were minimal; hence, there was a lot of trial and error in terms of what to use, how long to use for, and when to use. This added greatly to both the child and his family’s stress as, despite all efforts, the progress was painful and slow to nonexistent.

His contractures were by far the most life changing and distressing aspect of the disease. For an able-bodied child to survive cancer three times but lose his mobility, independence, and ultimately his life as a result of the treatment is hard to accept. To be under the care of so many providers, most of whom did not understand that the disease was extremely frightening, added to the pressure as it meant the family felt they had to do their own research and investigations. The untimely decisions; contradiction of self and others; complete inertia at times; constant running to occupational therapists, physiotherapists, dieticians, respiratory therapists, plastic surgeons, orthopedic surgeons, and ophthalmologists; and getting pulmonary tests and photopheresis were frustrating and confusing. In hindsight, the family was in a palliative situation at home for almost a year, unbeknownst to them.

## Discussion and conclusion

The main issue in this child’s journey seemed to be a lack of in-depth knowledge on steroid-refractory chronic GVHD among some healthcare professionals; the importance of being proactive, the vital role of a multidisciplinary team, and the contradictory advice provided to the patients when it came to medication, nutrition, and splints are also other issues. Despite multiple MRIs confirming ongoing inflammation and edema throughout his body, decisions on the course of action appeared either slow or ineffective.

Furthermore, the most distressing element of the child treatment journey was the lack of a clear and structured pathway multidisciplinary approach and the lack of education and resources for the parents and patients from the healthcare professionals, which led to several points of complete inertia in the progress of this patient. At one point, he saw seven consultants over four hospitals, resulting in a lot of travel and waiting in hospitals, which impacted home physiotherapy and nutritional goals. Each doctor reported this child’s condition differently, adding to the distress and confusion. Hematologists claimed ultimate responsibility for all decisions, although physiotherapists, occupational therapists, and plastic surgeons had become more important in the overall picture at one point. Rheumatologists, respiratory therapists, and hematologists appeared to work individually, and the parents certainly did not get the sense of a coordinated and strategic care plan, despite the team’s efforts to achieve this.

With no gastrointestinal GVHD, the child died malnourished, with words like anorexia being used to describe his state to the family, despite seeing so many consultants over so many hospitals. In the end, he faced the following challenges in his day-to-day life: inability to get in or out of bed independently or even roll over in bed, inability to perform self-care independently (bathing/washing, dressing, toileting), requiring assistance getting in and out of the car, difficulty in holding his head up for long periods, inability to run around or play, difficulty in managing 10 second walks, and inability to attend school.

### Areas of improvement and recommendations

- Who should be there? There was no central person or team controlling or coordinating his care. There was limited proactivity to reach out to other specialists, such as plastic surgeons, particularly concerning the musculoskeletal manifestations encountered. There was a lack of a strategic multidisciplinary plan controlled by a central source.
- There was a lack of “local” knowledge and experience in the management of chronic GVHD and extremely limited access to GVHD experts. For example, a simple episode of constipation that caused severe acute GVHD was overlooked for almost 3 months because of uncertainty and lack of knowledge.
- There were contradictions, confusion, and inertia among the medical community with regard to nutrition, steroid use, splint types, whether symptoms were even GVHD-related, and the general course of action.
- There was a lack of exposure/suggestions from the medical community to innovate and explore clinical trials. When this did happen, it was primarily led by parents who had invested significant time in researching these options.
- The reactive versus proactive approach stems from the lack of a plan. Even to the end, the child was treated too late. It was clear from the MRIs at the start of July that there were pleural effusions in his lungs, but this was not immediately acted upon.
- Absent multidisciplinary care: physiotherapy, occupational therapy, plastic surgery, dermatology, and others.

**Fig. 3:**
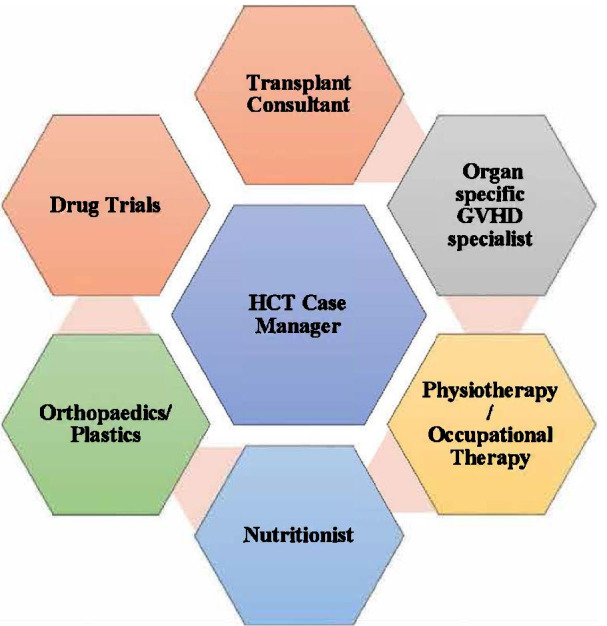
HSCT Hexagon

Based on this case experience, combined with the opinions of experts in the field and patient caretakers, we would like to recommend the transplant world to adapt or develop the “HSCT Hexagon” approach (Fig. 3) to help enhance patient and caregiver experience and for a smooth journey involving a multidisciplinary team making informed decisions in patients’ best interests.

#### HSCT Hexagon

We recommend that each transplant center should have a dedicated HSCT case manager who will be responsible for overseeing the patient’s journey from pre-transplant to up to 3 years. The HSCT case manager should have fast-track access to specialist services, be able to communicate directly with the service providers, and coordinate appointments for the patients. We suggest that the HSCT case manager is from one of the allied healthcare teams, that is, physiotherapists, occupational therapists, or nursing staff, as the majority of post-HSCT patients tend to have ongoing contact with this group of healthcare professionals. With the appropriate extended training, they can make swift recommendations in the best interest of the patient. Perhaps there is even a strong case for developing a role similar to that of the first contact practitioner (FCP) for GVHD/HSCT patients [9].

**Fig. 4:**
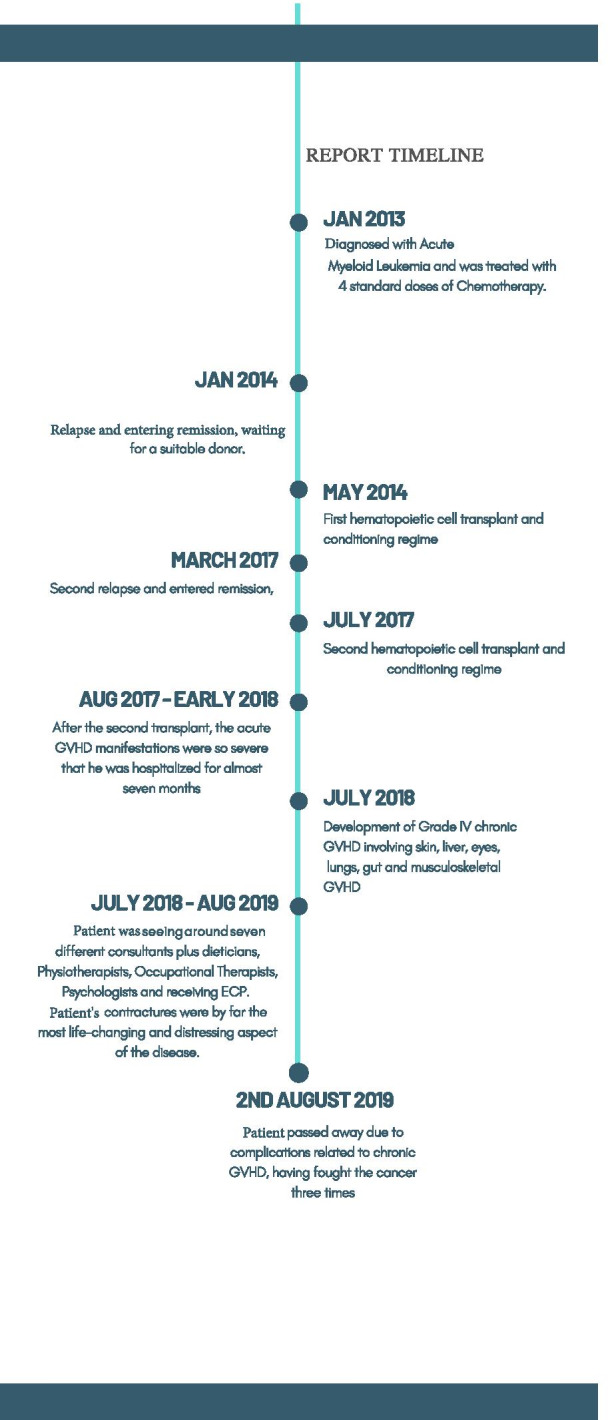
The child's treatment journey

Since the child died in August 2019, the treating hospital has since appointed a Bone Marrow Transplant Nurse Specialist. The family believes that this role may not have saved their child's life, but it would have significantly eased his physical and emotional suffering and taken a huge amount of stress and pressure away from day-to-day care (Fig. 4). No parent should have to research, lobby, and network worldwide and locally for appropriate care and medication, while caring for an extremely sick child.

Finally, transplant centers have a duty of care to each of their patients. A proactive approach with a well-defined pathway is needed for managing post-transplant complications and reducing the stress and anxiety of the patients and their caregivers. Patients should not be left on their own to experience the complications of their illness, have to flag the consultant, and wait in long queues before receiving specialist services. HSCT managers can be instrumental in maintaining communication between various specialties to help achieve optimal gains for patients. A universal pathway needs to be formulated in which the patient and the caretakers are aware of the prognosis and the action required in case of disease progression.

## Supplementary Information

**Additional file 1: Appendix S1.** Follow-up report.

**Additional file 2: Appendix S2.** Range of motion assessment report.

**Additional file 3: Appendix S3.** Skin assessment report.

## Data Availability

Data sharing not applicable to this article as no datasets were generated or analyzed during the current study.

## Abbreviations

HSCT: Hematopoietic stem cell transplantation
GVHD: Graft-versus-host disease
TPN: Total parenteral nutrition
ALT: Alanine aminotransferase
GGT: Gamma-glutamyl transferase
FAM: Fluticasone, azithromycin, and montelukast
ICU: Intensive care unit
CRP: C-reactive protein
