# Supplementary material for: Recommendations on service delivery to help reduce suffering and anxiety in patients and caregivers post-hematopoietic cell transplantation: a case report
Source: J Med Case Rep. 2021 Nov 5;15:549. doi: 10.1186/s13256-021-03126-4 (PMC8569970; doi:10.1186/s13256-021-03126-4)
Supplement: Supplementary file 1 — Additional file 1: Appendix S1. Follow-up report. [file 13256_2021_3126_MOESM1_ESM.pdf]

[redacted]

08/02/2019 MRI Pelvis

Indications: Leukaemia 2013. Bone marrow transplants x2. Graft-versus-host disease complicated by soft tissue inflammation, thickening. Scleroderma. Query tissues involved. Severe contractures.

Sequences: Coronal and axial TSE T1 and STIR.

Findings: Unfortunately, the presence of the contractures does result in the obliquity of the tissue to the planes of imaging. In addition, there is some degradation of image quality by movement artefact.

The visualised upper and lower limb apophyses and epiphyses are not yet fused.

Signal from the bone marrow of the vertebral bodies and sacrum is within normal limits.

No osteonecrosis has been demonstrated in the upper or lower limbs.

In the upper limbs, there are effusions in both glenohumeral and elbow joints. Signal from the scapulae is normal.
Within the limitations of the respiratory motion artefact, signal from the ribs is felt to be within normal limits.
There is patchy bone marrow oedema in both humeri.

There is quite marked oedema within the intercostal musculature and also involving the shoulder girdle muscles.
There is fluid signal both superficial and deep to the subscapularis muscles.
There is also oedema within the musculature of both upper arms and forearms.
Into fascia oedema is present around the brachial plexus on each side.

In the lower limbs, there are bilateral hip joint effusions. The knees are outside the field of view.
Oedema is present within both iliac bones and there are small fluid collections deep to both ilio psoas muscles.

Oedema is present within the short hip rotators bilaterally and also around the obturator internus muscles.

Further oedema / fluid is present deep to each gluteus minimus and also between the gluteus minimus and gluteus medius muscles on each side.

There is also oedema within the subcutaneous tissues around the shoulder girdles, thorax and abdomen.

Conclusion: Some degradation of image quality by movement artefact. Nonspecific but relatively symmetrical joint effusions. Bone marrow, muscle, subcutaneous tissue, inter muscular/ fascial oedema as described particularly around the shoulder girdles and pelvis with probable bursal fluid collections around the subscapularis and ilio psoas muscles. Overall, the appearances are most likely those of an inflammatory process involving predominantly the soft tissues.
