# Supplementary material for: Recommendations on service delivery to help reduce suffering and anxiety in patients and caregivers post-hematopoietic cell transplantation: a case report
Source: J Med Case Rep. 2021 Nov 5;15:549. doi: 10.1186/s13256-021-03126-4 (PMC8569970; doi:10.1186/s13256-021-03126-4)

**JOINTS AND FASCIA**<br><br>P-ROM score<br>*(see below)*<br>Shoulder (1-7) 2<br>Elbow (1-7) 4<br>Wrist/finger (1-7) 2<br>Ankle (1-4) 2	☐ No symptoms	☐ Mild tightness of arms or legs, normal or mild decreased range of motion (ROM) AND not affecting ADL	☐ Tightness of arms or legs OR joint contractures, erythema thought due to fasciitis, moderate decrease ROM AND mild to moderate limitation of ADL	☑ Contractures WITH significant decrease of ROM *AND* significant limitation of ADL (unable to tie shoes, button shirts, dress self etc.)

☐ *Abnormality present but explained entirely by non-GVHD documented cause (specify):* ______

☐ *Abnormality thought to represent GVHD PLUS other causes (specify):* ______

**GENITAL TRACT**<br>*(See Supplemental figure‡)*<br>☐ Not examined<br>*Currently sexually active*<br>☐ Yes<br>☑ No	☑ No signs	☐ Mild signs‡ and females with or without discomfort on exam	☐ Moderate signs‡ and may have symptoms with discomfort on exam	☐ Severe signs‡ with or without symptoms

☐ *Abnormality present but explained entirely by non-GVHD documented cause (specify):* ______

☐ *Abnormality thought to represent GVHD PLUS other causes (specify):* ______

Other indicators, clinical features or complications related to chronic GVHD (check all that apply and assign a score to severity (0-3) based on functional impact where applicable none – 0, mild -1, moderate -2, severe – 3)

☐ Ascites (serositis) ____	☐ Myasthenia Gravis ____	
☐ Pericardial Effusion ____	☐ Peripheral Neuropathy ____	☐ Eosinophilia > 500/μl ____
☑ Pleural Effusion(s) [illegible] 2	☐ Polymyositis ____	☐ Platelets < 100,000/μl ____
☐ Nephrotic syndrome ____	☑ Weight loss >5%* without GI symptoms	☐ Others (specify): ______

Biopsy obtained: ☐ Yes ☑ No **Organ biopsied:** ______ **GVHD confirmed by histology:** ☐ Yes ☑ No

Overall GVHD Severity
(Opinion of the evaluator) ☐ **No GVHD** ☐ **Mild** ☐ **Moderate** ☑ **Severe**

Change from prior evaluations: ☐ **No prior or current GVHD** ☐ **Improved** ☑ **Stable** ☐ **Worse** ☐ **N/A (baseline)**

Photographic Range of Motion (P-ROM):

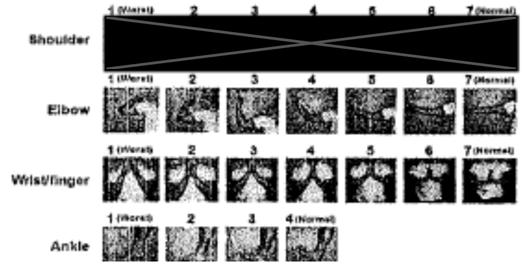

Supplement: Supplementary file 2 — Additional file 2: Appendix S2. Range of motion assessment report. [file 13256_2021_3126_MOESM2_ESM.pdf]
