# Supplementary material for: Recommendations on service delivery to help reduce suffering and anxiety in patients and caregivers post-hematopoietic cell transplantation: a case report
Source: J Med Case Rep. 2021 Nov 5;15:549. doi: 10.1186/s13256-021-03126-4 (PMC8569970; doi:10.1186/s13256-021-03126-4)
Supplement: Supplementary file 3 — Additional file 3: Appendix S3. Skin assessment report. [file 13256_2021_3126_MOESM3_ESM.pdf]

SCLERODERMA SKIN ASSESSMENT
(Modified Rodnan Skin Score)

Calculate a Modified Rodnan Skin Score by summing the scores from all evaluated anatomic areas.

A. Evaluate skin thickness by clinical palpation:

0 = normal skin thickness
1 = mild skin thickness
2 = moderate skin thickness
3 = severe skin thickness (inability to pinch skin into a fold)

B. Surface of anatomic areas evaluated (N = 17)

		Dates:	21/6/19				
**Area of Body**		**Range**	**Score**	**Score**	**Score**	**Score**	**Score**
Face		0-3	1				
Anterior Chest		0-3	3				
Abdomen		0-3	2				
Fingers	R	0-3	2				
	L	0-3	2				
Dorsium of Hands	R	0-3	2				
	L	0-3	2				
Forearms	R	0-3	3				
	L	0-3	3				
Upper arms	R	0-3	3				
	L	0-3	3				
Thighs	R	0-3	2				
	L	0-3	2				
Lower legs	R	0-3	2				
	L	0-3	2				
Dorsium of Feet	R	0-3	1				
	L	0-3	1				
TOTAL		0-51	36				
